# BugBuster: a novel automatic and reproducible workflow for metagenomic data analysis

**DOI:** 10.1093/bioadv/vbaf152

**Published:** 2025-06-26

**Authors:** Francisco Fuentes-Santander, Carolina Curiqueo, Rafael Araos, Juan A Ugalde

**Affiliations:** Center for Bioinformatics and Integrative Biology, Facultad de Ciencias de la Vida, Universidad Andres Bello, Santiago 8370186, Chile; Center for Bioinformatics and Integrative Biology, Facultad de Ciencias de la Vida, Universidad Andres Bello, Santiago 8370186, Chile; Genomics & Resistant Microbes Group (GeRM), Instituto de Ciencias e Innovación en Medicina (ICIM), Facultad de Medicina, Clínica Alemana, Universidad del Desarrollo, Santiago 7610658, Chile; Center for Bioinformatics and Integrative Biology, Facultad de Ciencias de la Vida, Universidad Andres Bello, Santiago 8370186, Chile

## Abstract

**Summary:**

Metagenomic sequencing generates massive datasets that capture the complete genetic content of a sample, enabling detailed characterization of microbial communities. Yet the software and processes necessary to transform raw data into biologically meaningful results have become increasingly complex, limiting accessibility for researchers without specialist expertise. In this work, we present a novel modular a reproducible workflow developed to facilitate the analysis of metagenomic data. BugBuster is a fully containerized, modular, and reproducible workflow implemented in Nextflow. The pipeline streamlines analysis at level of reads, contigs, and metagenome-assembled genomes, offering dedicated modules for taxonomic profiling and resistome characterization. Thanks to the use of containers, BugBuster can be deployed with minimal configuration on workstations, high-performance clusters, or cloud platforms. Together, these features allow the robust, scalable, and reproducible analysis of metagenomic datasets.

**Availability and implementation:**

BugBuster was written in Nextflow-DSL2. The program applications, user manual, example data and code are freely available at https://github.com/gene2dis/BugBuster.

## 1 Introduction

Metagenomics has established itself as a fundamental tool in various fields of microbiology, including pathogen identification, antimicrobial resistance monitoring, and industrial process optimization (Bashir *et al.* 2014).

Processing a metagenomic sample involves multiple steps, such as removing low-quality sequences and contaminants, taxonomic profiling, genome assembly, gene annotation, and binning. While various tools exist to perform each step, their outputs and standards are often incompatible or not directly comparable, adding complexity to the analysis of metagenomic datasets (Tamames and Puente-Sánchez 2018). The large volumes of data generated require advanced computational tools and expertise in high-performance infrastructure to run large-scale analyses efficiently. Numerous pipelines and workflows have been developed to process microbial data following specific protocols for different analytical stages (Navgire *et al.* 2022). Nevertheless, experienced users frequently assemble custom hybrid workflows by selecting analyses that best fit their requirements, adding complexity and challenges to reproducibility.

Despite their flexibility, these hybrid approaches pose significant challenges due to the lack of standardized interoperability between tools, requiring manual adjustments to integrate inputs and outputs. Additionally, the various and evolving dependencies associated with these workflows make them challenging to implement, even for experienced users, and are nearly inaccessible to beginners, particularly in unfamiliar computational environments (Kesh and Raghupathi 2004). Updating these workflows to incorporate new methodologies often requires rewriting scripts, further complicating maintenance and usability.

In addition to these concerns regarding interoperability and usability, reproducibility has become a key issue in computational biology. Publishing data and scripts on platforms such as GitHub or GitLab is becoming standard practice and even a requirement in some cases. However, making these resources available does not guarantee reproducibility because platform-specific dependencies can hinder deployment across different environments (Baykal *et al.* 2024). In addition, differing versions of libraries and tools on High-Performance Computing (HPC) systems can further complicate reproducibility.

A potential solution to these challenges is the use of container applications, such as Docker. These applications allow users to run applications in isolated environments by packaging all the necessary components, including files, libraries, and operating systems (Docker 2020). When combined with a workflow management system, such as Nextflow, these containers enable the creation of automated, scalable, reproducible, efficient, and portable workflows (Di Tommaso *et al.* 2017).

To address these challenges, we present BugBuster, a fully automated workflow for metagenomic data processing that covers all stages of analysis, from initial quality control to resistome detection and characterization. BugBuster was developed in Nextflow using the DSL2 syntax, offering a modular and flexible structure. Each process is encapsulated in Docker containers to ensure easy installation and high reproducibility. Additionally, it includes specialized modules for identifying antibiotic resistance genes that can be directly associated with specific taxa.

## 2 BugBuster pipeline

**Figure 1.**
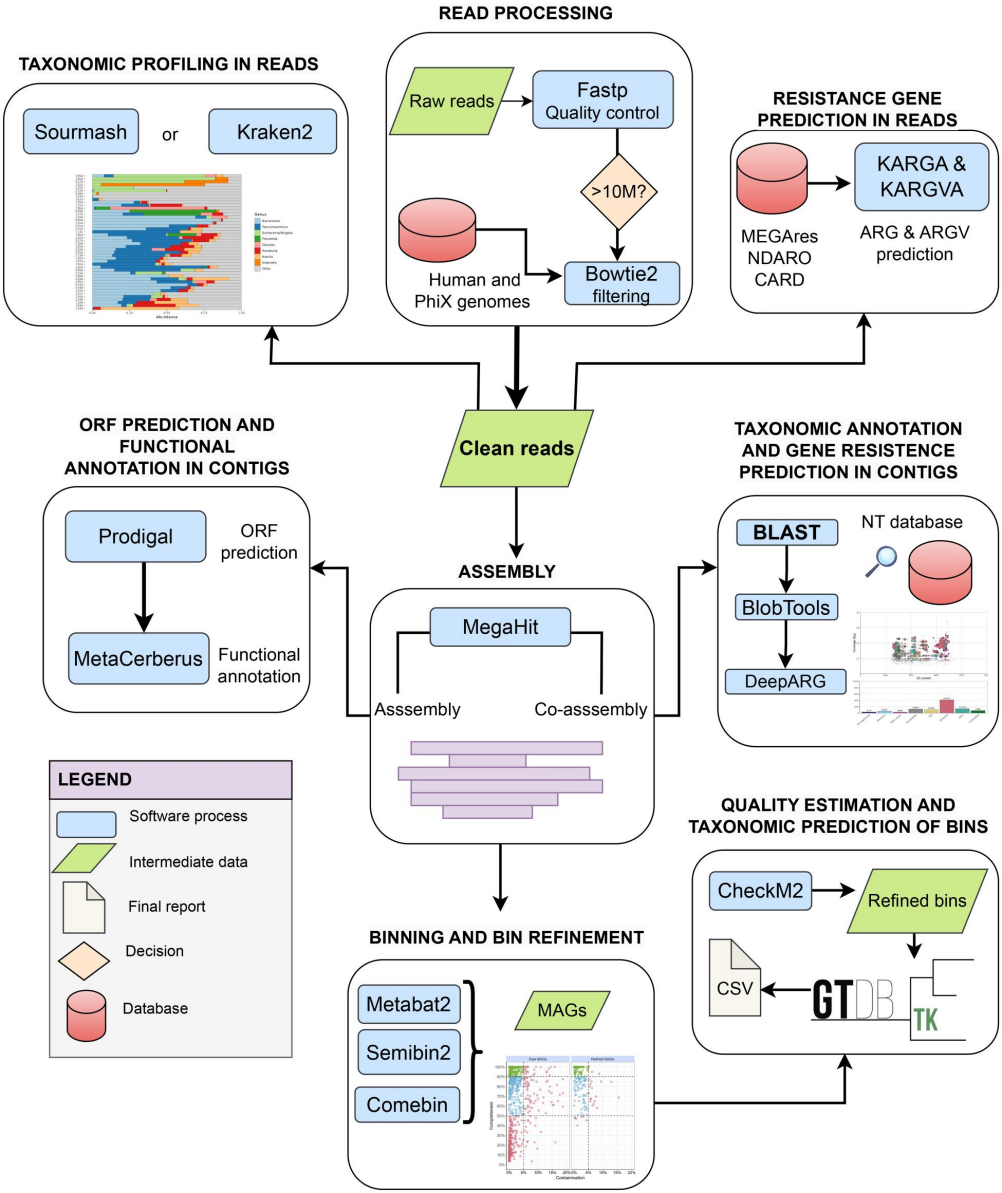
Workflow of the modules and customizable decisions during execution. The execution includes: (1) Quality filtering of reads; (2) Filtering of samples containing a minimum number of reads specified by the user; (3) Filtering of contaminant reads; (4) Prediction of antibiotic resistance genes and gene variants causing resistance at the read level; (5) Normalization of predicted genes by estimating the number of cells; (6) Taxonomic prediction and abundance estimation at the read level; (7) Reports for tracking reads and taxonomic reports; (8) Read assembly; (9) Taxonomic annotation of contigs; (10) Functional annotation of contigs; (11) ORF prediction in contigs; (12) Prediction of resistance genes at the contig level; (13) Contig report and two-dimensional scatter plots; (14) Contig filtering; (15) Metagenomic binning; (16) Refinement of assembled genomes in metagenomes (MAGs); (17) Prediction of MAG quality; (18) Taxonomic prediction of MAGs; (19) MAG results report.

BugBuster is written in Nextflow using the DSL2 syntax, which allows for a modular pipeline structure (Fig. 1). It consists of 61 processes that facilitate customization during execution. These 61 processes can be grouped into six steps that address metagenome processing: (i) Reads processing, (ii) Taxonomic profiling reads, (iii) Prediction of antibiotic resistance genes (ARGs) and resistance-causing gene variants (ARGVs) in reads, (iv) Assembly; (v) Binning; (vi) Taxonomy annotation and functional prediction in contigs. Some of the customization options currently available are:

- Assembly mode: Assembly, all metagenomes were processed individually; co-assembly and metagenomes were grouped, and a single assembly was performed.
- Software used for taxonomic profiling: Kraken2 (Wood *et al.* 2019) and Sourmash (Titus Brown and Irber 2016).
- Prediction of resistance genes and gene variants that cause antibiotic resistance in reads.
- Functional annotation, taxonomic prediction, and resistance gene predictions from contigs.
- Metagenomic binning

### 2.1 Description of BugBuster modules

#### 2.1.1 Read processing

Read quality filtering is performed using FastP v. 0.23.2 (Chen *et al.* 2018) with the following parameters—unqualified_percent_limit = 10,—cut_front,—cut_front_window_size = 4,—cut_front_mean_quality = 20,—cut_right,—cut_right_window_size = 4,—cut_right_mean_quality = 20,—detect_adapter_for_pe,—n_base_limit = 5 and—trim_poly_g. These parameters set by default in Bugbuster, but can be modified by the user. Subsequently, samples containing the minimum number of reads specified by the user are filtered. Additionally, host-contaminating reads can be discarded by mapping against a reference genome. By default, Bugbuster uses the human reference genome human reference genome T2T-CHM13v2.0 (Accession number: GCA_000001405.1) and the PhiX genome (Accession number: NC_001422) with Bowtie2 v. 2.5.3 (Langmead and Salzberg 2012) using the parameters -N = 1, -L = 20, -score-min=’G, 15, 6’, -R = 2, -i ‘S, 1, 0.75’. Data from the filtered reads during all steps is collected using a Bash script, and a report and plots are generated using an R script.

#### 2.1.2 Taxonomic profiling of metagenomic reads taxonomic profiling of metagenomic reads

Kraken2 workflow: Taxonomic prediction and abundance estimation at the read level are performed using Kraken2 v. 2.1.3 in conjunction with Bracken v. 2.9 (Lu *et al.* 2017). The results generated by Bracken are unified using Kraken-Biom v. 1.2.0 (Dabdoub 2016). Subsequently, the generated file in the biom format is transformed into a Phyloseq object (McMurdie and Holmes 2013).

*Sourmash workflow:* An alternative to Kraken2, is Sourmash, which uses MinHash sketches to represent the taxonomic signatures of large sequence sets. This approach allows for more efficient storage, reduces RAM and CPU usage, and enables more extensive reference databases. Taxonomic prediction and abundance estimation are performed using Sourmash v. 4.8.11. Estimation of the k-mer content in the reads is performed using the Sourmash sketch dna function with different parameters depending on the requested taxonomic resolution: Genus: -p k = 21, scaled = 1000, abund; Species: -p k = 31, scaled = 1000, abund; Strain: -p k = 51, scaled = 1000, abund. The minimum metagenome coverage estimation is then performed using the Sourmash gather function with its default parameters, changing the k-mer length according to the requested taxonomic resolution (Genus: k21; Species: k31; Strain: k51). Subsequently, the Sourmash tax annotation function is used to obtain the taxonomy assigned to the reads, and the generated file is used to create a Phyloseq object with an R script. The Kraken workflow supports taxonomic classification up to species level, while Sourmash workflow supports taxonomic classification up to strain level. The taxonomic level depends on the database used and user-defined parameters.

Finally, data on the proportion of taxonomically classified reads is collected for both workflows using a Bash script. These results are used to generate relative abundance plots with the microViz package v. 0.12.3 (Barnett *et al.* 2021) and bar plots showing the proportion of classified reads using ggplot2 v. 3.5.0.

#### 2.1.3 Resistance gene prediction on reads

Prediction of ARGs and ARGVs at the read level is performed with KARGA v. 1.02 (Prosperi and Marini 2021) and KARGVA v 1.0 (Marini *et al.* 2023), both using a kmer length of 17. The predicted ARG genes are filtered by 90% gene coverage or greater, while ARGV genes are filtered by 80% gene coverage or greater, and with at least 2 KmerSNPHits. Predicted genes are normalized by estimating the number of cells with ARGs-OAP v. 3.2.4 (Yin *et al.* 2023) using the default options. All generated results are unified in a report using an R script.

#### 2.1.4 Metagenome assembly

Read assembly is performed using MegaHit v. 1.2.9 (Li *et al.* 2015), in two different modes: per sample, where each metagenome is processed individually; and co-assembly, where all the samples are grouped, and a single assembly is performed. Contigs smaller than 1000 bp are filtered using BBmap version 39.06 (Bushnell 2014), and a report is generated with assembly metrics.

#### 2.1.5 Binning and bin refinement

Metagenome binning is performed using Metabat2 v. 2.15 (Kang *et al.* 2019), Semibin2 v. 2.1.0 (Pan *et al.* 2023) using the human-intestine trained model by default (modifiable by the user) and Comebin v. 1.0.4 (Wang *et al.* 2024) with three attempts, the first with the default options, followed by reduction of the embedding size in case there is a failure on the process, using the following parameters: -b 896, -e 1792, -c 1792 for the second attempt, and -b 512, -e 1024, -c 1024 for a final attempt. Subsequently, the metagenome-assembled genomes (MAGs) generated are refined with MetaWrap v.1.2 (Uritskiy *et al.* 2018), using default thresholds of a minimum completeness of 50% and a maximum contamination of 10%.

As a consideration for users, the current pipeline version does not perform MAG dereplication by default to preserve subtle genomic differences across samples. Future versions may incorporate this as an optional process. Metabat2 parameters and MetaWrap quality thresholds can be modified by the user through the configuration file. Currently, customization options are not available for Semibin2 and Comebin. The quality prediction of unrefined and refined bins is performed with CheckM2 v. 1.0.1 (Chklovski *et al.* 2024) using the default parameters. The refined bins are taxonomically classified using GTDB-TK v. 2.4.0 (Chaumeil *et al.* 2022) against the Genome Taxonomy Database (GTDB) release 220. Finally, the results are unified in a single Comma-separated values (CSV) file, which is used to generate quality and taxonomy graphs using an R script. In the co-ensemble mode, the coverage in each sample is also calculated separately for each refined bin using Bedtools v. 2.31.1 (Quinlan and Hall 2010) and Samtools v. 1.17 (Danecek *et al.* 2021), and the coverage data are unified with the taxonomy and quality data in a CSV file.

#### 2.1.6 Taxonomic annotation and functional prediction in contigs

Taxonomic annotation of contigs is initially performed using Blastn v. 2.15.0 (Altschul *et al.* 1990) against the NT database. The search results are used to assign taxonomy with BlobTools v.1.1.1 (Laetsch and Blaxter 2017) with the default parameters. Identification of antibiotic resistance genes at the contig level is performed with DeepARG v. 1.0.4 (Arango-Argoty *et al.* 2018) using the default parameters. The results generated with both software are unified in a CSV file and visualized with an R script. ORF prediction in contigs is performed using Prodigal v. 2.6.3 (Hyatt *et al.* 2010) with the metagenomics option. Functional annotation of the metagenomic assembly is performed using MetaCerberus v. 1.2.1 (Figueroa *et al.* 2024), utilizing, by default, the KOFam_all, COG, VOG, PHROG, and CAZy HMMs available in its database.

## 3 Tests dataset

To illustrate the results of the BugBuster pipeline, we used nine simulated samples of metagenomic data from the human gastrointestinal tract from the Critical Assessment of Metagenome Interpretation (CAMI) (Fritz *et al.* 2019). Using this data, we tested the pipeline with these options:—assembly_mode “assembly”—taxonomic_profiler “sourmash”—read_arg_prediction—contig_tax_and_arg—include_binning. All the data was processed on an 80-CPU Intel Xeon E7-4820 v4 server with 2TB RAM, but limiting the CPU usage to 20 cores. With this limitations, the processing time for the nine samples was close to 7 days.

## 4 Results

### 4.1 Read preprocessing

**Figure 2.**
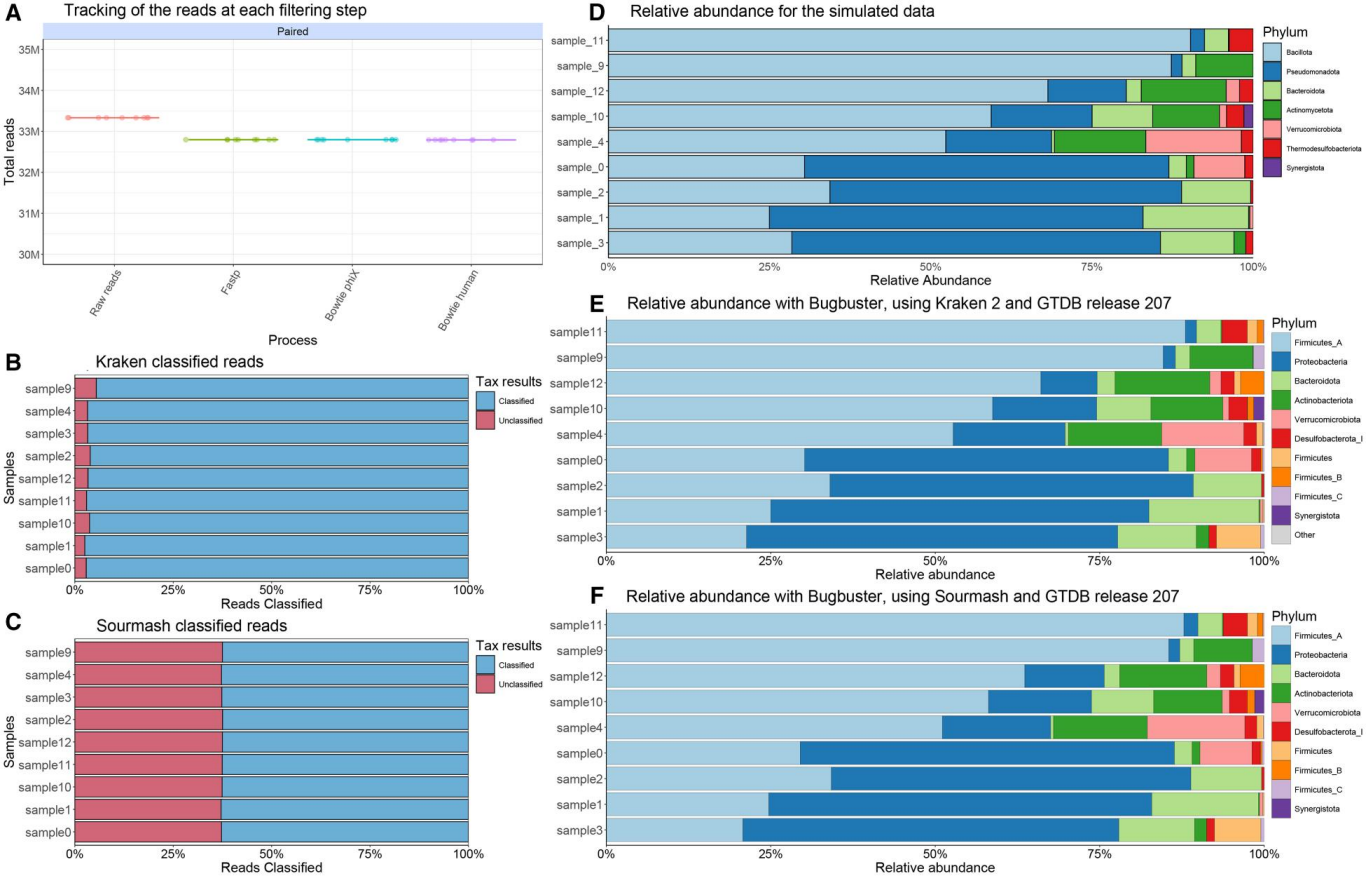
Summary of outputs from the initial data processing steps performed by BugBuster and the comparison of taxonomic classifications obtained using sourmash and Kraken2. (A) Tracking the reads at each filtering step. Bowtie PhiX and Bowtie Human represent the number of reads that passed the filtering for PhiX phage and human reads, respectively. (B) Reads classified with Kraken 2 with a confidence of 0.1 and GTDB release 207. (C) Reads classified with sourmash at species-level configuration and GTDB release 207. (D) Relative abundance of the simulated communities provided in the CAMI challenge. (E) Relative abundance with BugBuster, using Kraken 2 with a confidence of 0.1 and GTDB release 207. (F) Relative abundance with BugBuster, using Sourmash at species-level configuration and GTDB release 207.

The processing of simulated gut microbiota reads shows a progressive decrease in the total number of reads throughout the filtering steps. Based on quality criteria, 1.62% of reads were removed, and no human contamination was detected (Fig. 2A).

### 4.2 Taxonomic profiling and abundance estimation

The results show a comparison between Sourmash and Kraken using the GTDB release 207 database and the simulated data. On average, Kraken classified approximately 96.5% of the reads, whereas Sourmash classified approximately 62.5% (Fig. 2B and C). We kept the proportions of taxa in the two workflows with respect to the CAMI data set at the phylum level, observing differences only in the taxonomic names assigned by the respective databases due to different naming conventions between NCBI and GTDB (Fig. 2D–F). In particular, variations in the naming of taxonomic groups within the phylum Firmicutes are mainly due to the number of genomes included in the GTDB database and their detailed taxonomic classification at the strain level (Parks *et al*. 2022). As a result, the GTDB database assigns additional identifiers, such as letter codes, after the phylum name (e.g., Firmicutes_B) to distinguish the genomes of unique species.

### 4.3 Resistance gene prediction in reads

Processed reads can also be utilized to predict antibiotic resistance genes (ARGs) and their variants (ARGVs). For ARG prediction, we used the Megares v3.0 database (Bonin *et al.* 2023), identifying 824 genes in total (Supplementary Table S1, available as supplementary data at *Bioinformatics Advances* online). To identify ARG, we employed the KARGVA v5 database (Marini *et al.* 2023), which led to the identification of 358 predicted genes in total (Supplementary Table S2, available as supplementary data at *Bioinformatics Advances* online).

### 4.4 Assembly, taxonomic prediction, and resistance gene prediction in contigs

**Figure 3.**
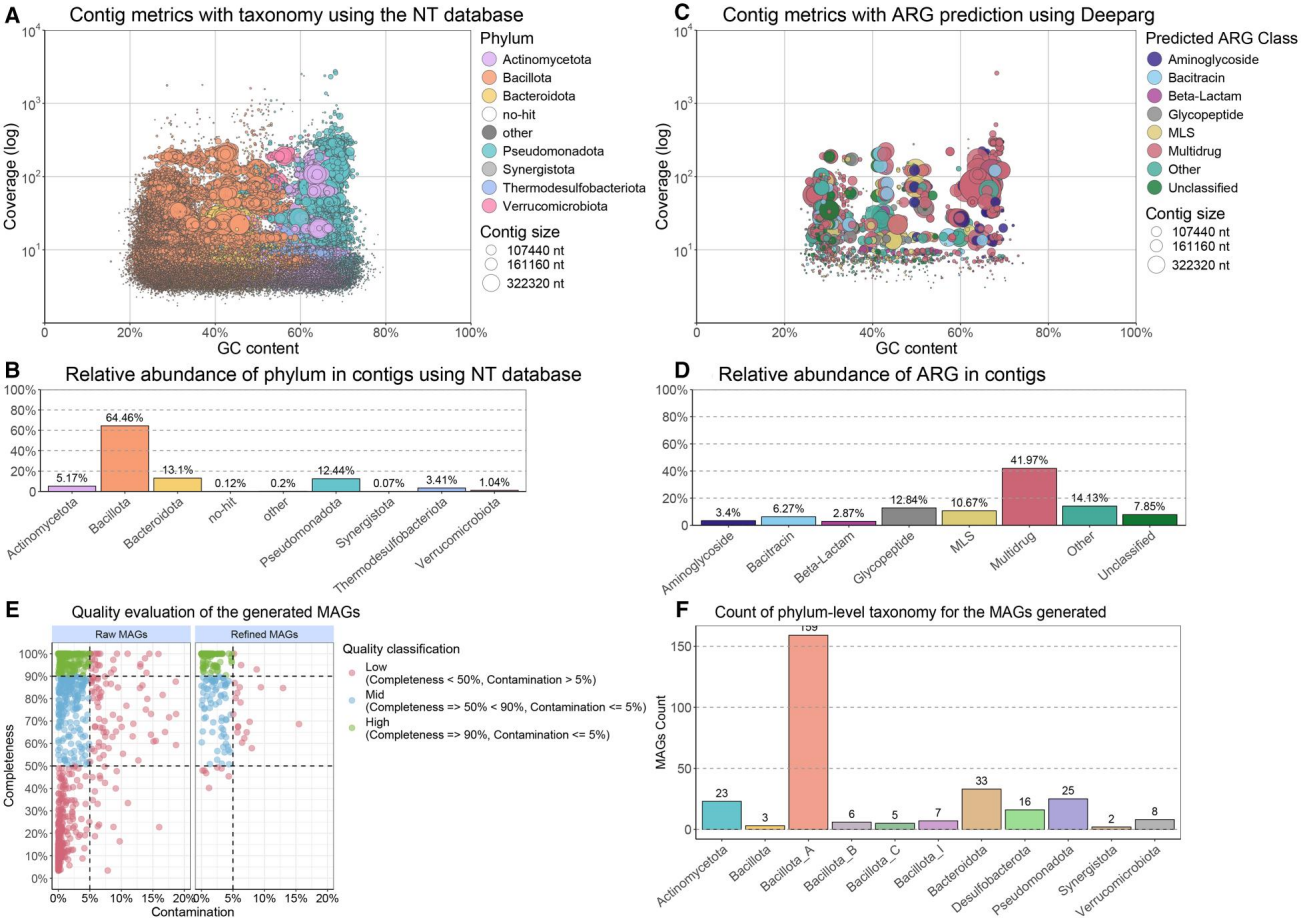
Summary of outputs from contig and MAGs processing steps performed by BugBuster. (A) Blobplot displaying contig metrics and taxonomy assigned using Blastn, Blobtools, and the NT database. (B) Relative abundance of taxonomy at the phylum level in the contigs, classified with Blastn, BlobTools, and the NT database. (C) Blobplot presenting contig metrics and ARGs predicted using Deeparg. (D) Relative abundance of ARGs identified in contigs using Deeparg. (E) Quality evaluation of the generated MAGs. Raw MAGs refer to the MAGs produced with Comebin, Semibin2, and Metabat2 across all samples. Refined MAGs represent the total number of refined MAGs reconstructed across all samples. (F) Overview of the phylum-level taxonomy of the MAGs generated for the entire sample set.

Each sample was assembled individually, and contigs larger than 1 KB were kept for further analysis. The results show the taxonomy of the contigs present in all samples using blobplots, we obtained an average N50 of 2.175 KB and taxonomically classified 99.88% of the generated contigs throughout the entire set of samples (Fig. 3A and B). The proportion of phyla in the contigs obtained by BugBuster was similar to that provided by the simulated CAMI data, with Bacillota being the most abundant phylum (Supplementary Table S3, available as supplementary data at *Bioinformatics Advances* online). All filtered contigs were used to search for resistance genes using DeepARG, identifying 1706 predicted genes throughout the entire set of samples (Fig. 3C and D).

### 4.5 Binning, quality estimation, and taxonomic prediction in bins

For binning, we obtained 112, 72, and 95 high-quality bins; 61, 66, and 73 medium-quality bins; and 221, 135, and 169 low-quality bins from Comebin, MetaBAT2, and SemiBin2, respectively. This set of bins was used to generate 115 high-quality, 73 medium-quality, and 27 low-quality bins with MetaWRAP (Fig. 3E). In total, we reconstructed 215 MAGs of 266 genomes provided in the CAMI dataset. All these 215 MAGs were successfully classified taxonomically at species level (Fig. 3F).

## 5 Conclusions

BugBuster provides an easy-to-implement and highly reproducible workflow covering pre-processing, taxonomic classification, antibiotic resistance gene prediction, assembly, and MAG refinement. It includes documentation for users (https://github.com/gene2dis/BugBuster) and generates visual outputs to better interpret the results. BugBuster offers modular flexibility, allowing users to select specific modules and optimize configurations for their research needs. The pipeline will be continuously updated to integrate the latest analysis methods. Its DSL2-based modularity enables the efficient incorporation of new tools, including future enhancements for mobile genetic element detection, resistance gene clustering, and optimized execution on limited computational resources.

## Supplementary Material

vbaf152_Supplementary_Data

## Code availability

All code is available at https://github.com/gene2dis/BugBuster
